# Genomics-assisted breeding in four major pulse crops of developing countries: present status and prospects

**DOI:** 10.1007/s00122-014-2301-3

**Published:** 2014-04-08

**Authors:** Abhishek Bohra, Manish K. Pandey, Uday C. Jha, Balwant Singh, Indra P. Singh, Dibendu Datta, Sushil K. Chaturvedi, N. Nadarajan, Rajeev K. Varshney

**Affiliations:** 1 Indian Institute of Pulses Research (IIPR), Kanpur, 208024 India; 2 International Crops Research Institute for the Semi-Arid Tropics (ICRISAT), Hyderabad, 502324 India; 3 National Research Centre on Plant Biotechnology (NRCPB), New Delhi, 110012 India; 4 The University of Western Australia (UWA), Crawley, 6009 Australia

## Abstract

*****Key message***:**

**Given recent advances in pulse molecular biology, genomics-driven breeding has emerged as a promising approach to address the issues of limited genetic gain and low productivity in various pulse crops.**

**Abstract:**

The global population is continuously increasing and is expected to reach nine billion by 2050. This huge population pressure will lead to severe shortage of food, natural resources and arable land. Such an alarming situation is most likely to arise in developing countries due to increase in the proportion of people suffering from protein and micronutrient malnutrition. Pulses being a primary and affordable source of proteins and minerals play a key role in alleviating the protein calorie malnutrition, micronutrient deficiencies and other undernourishment-related issues. Additionally, pulses are a vital source of livelihood generation for millions of resource-poor farmers practising agriculture in the semi-arid and sub-tropical regions. Limited success achieved through conventional breeding so far in most of the pulse crops will not be enough to feed the ever increasing population. In this context, genomics-assisted breeding (GAB) holds promise in enhancing the genetic gains. Though pulses have long been considered as orphan crops, recent advances in the area of pulse genomics are noteworthy, e.g. discovery of genome-wide genetic markers, high-throughput genotyping and sequencing platforms, high-density genetic linkage/QTL maps and, more importantly, the availability of whole-genome sequence. With genome sequence in hand, there is a great scope to apply genome-wide methods for trait mapping using association studies and to choose desirable genotypes via genomic selection. It is anticipated that GAB will speed up the progress of genetic improvement of pulses, leading to the rapid development of cultivars with higher yield, enhanced stress tolerance and wider adaptability.

## Introduction

The Fabaceae/Leguminosae or legume family with ~20,000 species is the third largest family in the plant kingdom and second most important after Gramineae or Poaceae as mainstays for human food/protein security (Cannon et al. 2009; Gepts et al. 2005; Weeden 2007; Young et al. 2003). Legumes are endowed with the unique property of biologically fixing atmospheric nitrogen via symbiosis, making them an integral component of sustainable agricultural production systems (Zhu et al. 2005). In the Fabaceae, grain legumes or pulses are particularly important in supplying adequate quantity of lysine-rich protein to humans, thereby complementing the conventional cereal-based carbohydrate-rich diets, which are otherwise deficient in lysine and tryptophan (Broughton et al. 2003; Ufaz and Galili 2008). Additionally, pulses are potential sources of several essential minerals, vitamins and secondary metabolites like isoflavonoids in human diets (Cannon et al. 2009). Concerning protein deficiency, it is important to emphasize that globally over one billion people are currently suffering from protein and micronutrient malnutrition (Godfray et al. 2010). In this context, pulses by virtue of their high protein, vitamin and mineral content play a crucial role in alleviating micronutrient deficiencies, undernourishment or protein calorie malnutrition (PCM), especially in the less-developed countries (Broughton et al. 2003).

FAO categorizes only those legumes as *pulses* which are harvested exclusively for grain purpose, thereby recognizing a total of 11 pulse crops (http://faostat.fao.org/; Akibode and Maredia 2011). In terms of worldwide pulse production, a total of 70.41 million tons (m t) are harvested annually from 77.5 million (m) ha area with a productivity of 907 kg/ha (FAOSTAT 2012). Almost 90 % of the global pulse production (62.98 m t) is shared by major pulse crops, viz. dry beans (mainly common bean), chickpea, dry peas (pea), cowpea, pigeonpea, lentil and faba bean. Based on their adaptability to tropical and temperate agro-climatic conditions, these pulse crops can be further categorized into two distinct groups, i.e. (1) warm season crops (common bean, pigeonpea and cowpea) and (2) cool season crops (pea, chickpea, lentil and faba bean) (Cannon et al. 2009; Young et al. 2003; Zhu et al. 2005). Interestingly, chickpea, pea and lentil are among the founder grain crops, which experienced domestication early in pre-history (c. 11,000 years ago), and these paved the way for establishment of modern agriculture (Zohary and Hopf 2000). The pulse crops have always been a key contributor to maintaining sustainability of the farming systems in the semi-arid and sub-tropical world and in generating livelihood and food security to millions of resource-poor people inhabiting these regions (Broughton et al. 2003).

**Fig. 1:**
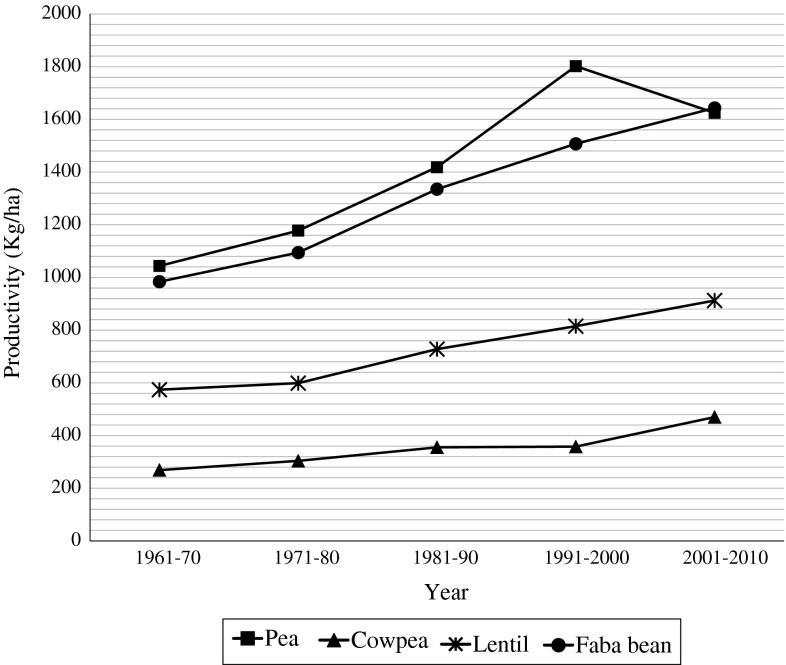
Global trends in productivity of four major pulse crops. The figure illustrates trends in productivity of major pulse crops witnessed over the last five decades

Owing to their immense agricultural value, exhaustive research has been done for pulse improvement through conventional breeding, resulting in the development and release of several high-yielding varieties (Gaur et al. 2012; Pérez de la Vega et al. 2011; Saxena 2008; Singh 2005; Torres et al. 2011), followed by an increase in the global area under pulses from 64 to 77.5 m ha over the last 50 years (FAOSTAT 2012). With respect to productivity, however, appreciable gains have not been materialized so far in any of the major pulse crops (Fig. 1). The productivity of major pulse crops remains dismally low, around 1,000 kg/ha, and large gap exists between their potential and actual yields (FAOSTAT 2012; Varshney et al. 2013a). In this context, integrating genomic tools with conventional breeding methods holds the key to accelerate the progress of crop improvement. Unlike cereals like wheat and barley (which were domesticated almost at the same time as pulses), limited efforts have been directed towards undertaking molecular breeding or more appropriately genomics-assisted breeding (GAB) of pulse crops (Muchero et al. 2009a; Muehlbauer et al. 2006; Timko et al. 2007; Varshney et al. 2010). One likely reason is the limited attention of the international research community to these pulse crops. As a result, there has been a dearth of prerequisite genomic tools to commence GAB at a larger level (Varshney et al. 2009a). These crops, therefore, are often referred to as “orphan crops”. Nevertheless, in some pulse crops, large-scale genomic tools, technologies and platforms have become available in recent years (Gaur et al. 2012; Gepts et al. 2008; Kelly et al. 2003; Muehlbauer et al. 2006; Rubiales et al. 2011; Varshney et al. 2013a), thereby opening up new avenues for practising GAB. This is a highly opportune time for reframing our breeding strategies, allowing judicious and routine use of genomic tools for genetic enhancement of modern cultivars as well as diversification of the primary gene pool through introduction of desirable alien alleles from crop wild relatives (CWRs). Advances in genomics and molecular breeding have been discussed in details for chickpea and pigeonpea in some recent reviews (Varshney et al. 2013a). However, not much information is available about recent developments in case of other pulse crops. In consideration of the above, this review summarizes the production scenario and constraints, the available genomic resources and their downstream applications as well as prospects for GAB in four selected pulse crops, i.e. cowpea (*Vigna unguiculata* (L.) Walp.), pea (*Pisum sativum* L.), lentil (*Lens culinaris* Medik.) and faba bean (*Vicia faba* L.).

## Global production scenario and major yield constraints

Although there are several warm and cool season pulse crops that make important portion of diets of the poor in developing countries, four major pulse crops, namely, cowpea, pea, lentil and faba bean, have been included here for discussion.

### Cowpea

Cowpea (*Vigna unguiculata* (L.) Walp.), also referred to as black-eyed pea, crowder pea or lobia, is a self-pollinating diploid (2*n* = 2*x* = 22) species with an estimated genome size of 620 Mb (Chen et al. 2007; Singh 2005). It is an important warm season grain legume cultivated in ~30 countries (Singh 2005). Interestingly, more than 80 % of dry cowpea produce comes from three countries (Niger, Nigeria and Burkina Faso) of West Africa that cover nearly 83 % of the global cowpea area (FAOSTAT 2012; Popelka et al. 2006). Therefore, cowpea remains the primary source of income for small-scale farmers practising agriculture in dry Savannah of sub-Saharan Africa. Furthermore, cowpea also provides a cheap and highly nutritious feed for livestock in tropical West and Central Africa (Kamara et al. 2012). Asparagus bean (also known as snake bean or yardlong bean) is another cultivar group (cv.-gr. *sesquipedalis*) of cowpea that reflects remarkable morphological variations from African cowpea (cv.-gr. *unguiculata*) in plant architecture, growth habit and various pod-/seed-related characters (Kongjaimun et al. 2013; Singh 2005; Timko et al. 2007; Xu et al. 2013). Asparagus bean is grown primarily in Southeast and East Asia for its very long and tender pods, which are harvested at the immature stage and considered a highly nutritious vegetable (Xu et al. 2010, 2011a, b, 2012a).

Globally, cowpea has shown an increasing trend in its cultivation area from 2.41 m ha to 10.68 m ha over the last five decades (FAOSTAT 2012). The miserably low productivity of cowpea (~470 kg/ha) is largely attributable to a variety of constraints that prevail in cowpea-growing areas including diseases such as bacterial blight (*Xanthomonas axonopodis* pv. *vignicola* (Burkh.) Dye), rust (*Uromyces phaseoli* var. *vignae* Barclay), *Sphaceloma* scab (*Elsinoe phaseoli* Jenkins) and leaf spot (*Septoria vignicola* Rao), and insects/pests such as legume flower thrips (*Megalurothrips sjostedti* Trybom), pod borer (*Maruca vitrata* Fabricius) and storage weevil (*Callosobruchus maculatus* Fabricius) (Singh 2005). Apart from the above-mentioned constraints, instances of severe parasitism by weeds (*Striga gesnerioides* (Willd.) Vatke and *Alectra vogelii* (L.) Benth) resulting in 85–100 % loss have also been observed in cowpea (Kamara et al. 2012). The inherent tolerance to drought, heat and poor soil fertility makes cowpea an attractive crop for low-input farming systems in the Sudanian and Sahelian semi-arid regions of Africa (Hall et al. 2003; Hall 2004; Muchero et al. 2009a; Popelka et al. 2006). However, despite its high tolerance to drought, considerable reduction in cowpea yield has been reported due to prolonged drought periods in sub-Saharan Africa (Hall et al. 2003; Hall 2004; Muchero et al. 2009b).

### Pea

Pea (*Pisum sativum* L.) is a self-pollinating crop with 4,063 Mb genome organized into seven pairs of homologous chromosomes (2*n* = 2*x* = 14) (Arumuganathan and Earle 1991). Worldwide, a total of 9.86 m t of dry peas is harvested annually with exceptionally high productivity (1,558 kg/ha). The three major pea producers, i.e. Russian Federation, Canada and China, collectively contribute around 56 % (5.57 m t) and 54 % (3.39 m ha) to the global production and area, respectively (FAOSTAT 2012). Interestingly, no major antinutritional factor (ANF) has been reported in pea seeds, thereby making dry pea seeds a high-quality source for livestock feed and human consumption. Quite noticeably, almost half of the dry pea seeds harvested globally are used to feed livestock (Rubiales et al. 2011).

Among several biotic stresses affecting pea yields, *Fusarium* wilt (*F. oxysporum* f. sp. *pisi* (van Hall) Snyd. and Hans.), *Ascochyta* blight, a complex fungal disease caused by *Mycosphaerella pinodes* (Berk. and Blox.) Vestergr., *Phoma medicaginis* Malbr. and Roum. var. *pinodella* and *Ascochyta pisi* Lib.), root rot (*Aphanomyces euteiches* Drech.) and powdery mildew (*Erysiphe pisi* DC) are the most devastating diseases causing significant losses (Dixon 1987; Rubiales et al. 2011; Timmerman-Vaughan et al. 2002; Xue et al. 1997). In addition, one insect pest that has also emerged as a serious threat to pea production is pea aphid, *Acyrthosiphon pisum* (Harris), causing complete crop failure under conditions of severe infestations (Wale 2002).

### Lentil

Lentil (*Lens culinaris* Medik.) is a self-pollinated diploid (2*n* = 2*x* = 14) crop with a large genome size (4,063 Mb) (Arumuganathan and Earle 1991). From the standpoint of global production, lentil stands fifth with 4.55 m t being produced annually from an area of 4.24 m ha (FAOSTAT 2012). Major lentil-growing countries are India, Australia, Canada and Turkey, together producing more than 73 % of the world’s lentil (FAOSTAT 2012). Due to higher protein content and better digestibility, lentil contributes to nutritional and food security for the people in the northern temperate, Mediterranean and sub-tropical savannah regions (Sharpe et al. 2013).

Various fungal diseases affecting lentil yield substantially have been reported, which include *Ascochyta* blight (*A. lentis* Vassilievsky), *Fusarium* wilt (*F. oxysporum* f.sp. *lentis* Vasd. and Srin.), anthracnose (*C. truncatum* (Schwein.) Andrus and Moore), blight (*Stemphylium botryosum* Wallr.), rust (*Uromyces viciae*-*fabae* Pers.), collar rot (*Sclerotiun* *rolfsii* Sacc.), root rot (*Rhizoctonia* *solani* Kühn), dry root rot (*R. bataticola* Taub.) and white mould (*Sclerotinia sclerotiorum* (Lib.) de Bary) (Ford et al. 2007; Muehlbauer et al. 2006; Pérez de la Vega et al. 2011). Aside from biotic factors, lentil production is also vulnerable to temperature extremities including cold and heat stresses and others like drought and salinity (Muehlbauer et al. 2006).

### Faba bean

Faba bean (*Vicia faba* L.), also known as broad bean or horse bean, has six pairs of chromosomes and 13,000 Mb genome representing one of the largest genomes among legumes that is almost three times greater than pea and lentil (Cruz-Izquierdo et al. 2012; Yang et al. 2012; Young et al. 2003). It is cultivated in about 60 countries covering a total of 2.43 m ha area with an annual production of 4 m t (FAOSTAT 2012). Worldwide, China (0.95 m ha), Ethiopia (0.45 m ha), Morocco (0.18 m ha) and Australia (0.16 m ha) are the main faba bean-growing countries. China alone produces 35 % (1.4 m t) of the global dry faba beans followed by Ethiopia (0.71 m t) and Australia (0.42 m t). It is a dual-purpose crop, which not only provides inexpensive proteins for human consumption (particularly in western Asia and northern Africa), but also serves as a prime livestock feed in Europe and Australia (Alghamdi et al. 2012; Ellwood et al. 2008; Torres et al. 2006, 2011; Zeid et al. 2009).

Notwithstanding the higher productivity of faba bean (1,666 kg/ha), the global area under faba bean cultivation has declined over the last five decades (FAOSTAT 2012). Faba bean production is constrained by a number of biotic factors including fungal, bacterial and viral diseases, nematodes and pests (Gnanasambandam et al. 2012). Amongst various diseases, rust (*Uromyces viciae*-*fabae* (Pers.) J. Schröt.), chocolate spot (*Botrytis fabae* Stard.), *Ascochyta* blight (*A. fabae* Sperg.) and downy mildew (*Peronospora viciae* (Berk.) Caspary) are of considerable economic importance (Cubero and Nadal 2005; Gnanasambandam et al. 2012; Torres et al. 2006, 2011). Apart from the diseases mentioned above, zonate spot (*Cercospora zonata* Wint.), roo rot (*F. solani* Mart.) and blister disease (*Olpidium viciae* Kusano) also cause significant yield loss, particularly in China (Li-Juan et al. 1993; Saxena et al. 1993). In addition, the viral diseases that negatively affect faba bean production involve broad bean mosaic virus (BBMV), broad bean wilt virus (BBMV), turnip mosaic virus (TuMV), soybean mosaic virus (SMV) and cucumber mosaic virus (CMV) (Saxena et al. 1993). Among important insect pests, faba bean beetle (*Bruchus rufimanus* Boheman), medic aphid (*Aphis medicaginis* Koch and *Myzus persicae*) and root nodule weevil (*Sitona* *amurensis* Faust and *S*. *lineatus* L.) are the other damaging agents (Bardner 1983; Cubero and Nadal 2005; Li-Juan et al. 1993; Saxena et al. 1993). Moreover, frequent occurrence of a parasitic weed broomrape (*Orobanche crenata* Forks) often presents a great menace to faba bean cultivation in the Mediterranean region, North Africa and the Middle East (Díaz-Ruiz et al. 2009a; Rubiales and Fernández-Aparicio 2012; Torres et al. 2010) and several reports have documented yield loss up to 80 % (Gressel et al. 2004) or even complete crop failure (Sauerborn and Saxena 1986).

Besides biotic constraints, faba bean also suffers from drought and cold stresses, frost injury and presence of ANFs in seeds (Arbaoui et al. 2008; Torres et al. 2011). Therefore, to stabilize faba bean yield, development of genotypes exhibiting resistance to the above-mentioned biotic and abiotic stresses has always been a prime objective in faba bean breeding. Moreover, the partial cross-pollinating nature and existence of cytoplasmic genetic male sterility (CGMS) have steered faba bean breeding towards development of CGMS-based hybrids for exploitation of heterosis and enhancement of productivity (Bond 1989; Link et al. 1996, 1997).

## Genomic resources

Concerning pulse genomics, a rapid progress has been witnessed over the last 10 years generating a plethora of genomic tools for their extensive use in pulse improvement programmes. These resources include (1) different kinds of bacterial artificial chromosome (BAC)-derived resources like BAC libraries, BAC-end sequences (BESs), BAC-associated simple sequence repeat (SSR) markers (BES-SSRs) and physical maps; (2) genome-wide distributed molecular markers and automated genotyping platforms; and (3) the transcriptome and whole-genome assemblies.

### BAC-based resources

BAC libraries are valuable tools for facilitating various genetic applications such as DNA marker development, gene/QTL cloning, construction of physical map and BAC-to-BAC genome sequencing (Farrar and Donnison 2007). In pulses, several BAC/BIBAC libraries were established, providing extensive genome coverage in the respective crops, viz. cowpea (~9×) and pea (~2.2×) (Coyne et al. 2007; Kami et al. 2006). To date, however, no BAC libraries have been reported for lentil and faba bean. BAC libraries have been used for developing physical map and assembling the genome sequences. In this context, BACs are subjected to fingerprinting and these fingerprints are then used as seeds for the development of genome-wide physical maps and in the determination of minimum tiling path (MTP) for assembling the whole-genome sequence (Venter et al. 1996). A high-quality BAC-based physical map is now available for cowpea (790 contigs and 2,535 singletons, http://phymap.ucdavis.edu/cowpea/).

To enhance the accuracy of physical maps or assembling the sequences of BACs in the whole-genome sequencing, selected or entire set of BACs are also used for generating BESs. Additionally, the utility of these BESs in large-scale marker development has also been demonstrated through in silico SSR mining in cowpea (Xu et al. 2011a). These BES-associated markers such as BES-SSRs represent the potential anchoring points for integrating genome-wide physical maps with high-density genetic maps (Córdoba et al. 2010).

### Genome-wide distributed molecular markers

Starting from the introduction of hybridization based markers, viz. restriction fragment length polymorphism (RFLP), consistent improvements have been made in the area of DNA marker development and genotyping (see Bohra 2013). To this end, the traditional DNA marker technologies are being increasingly replaced by next-generation sequencing (NGS)-based high-throughput (HTP) discovery of DNA markers, especially single nucleotide polymorphisms (SNPs) (Varshney et al. 2009b). Further, on account of their amenability to automated genotyping platforms, SNPs have emerged as the preferred markers for next generation, substituting the earlier hybridization as well as polymerase chain reaction (PCR)-based assays (Varshney et al. 2009b). Through in silico mining of expressed sequence tags (ESTs), transcriptomes and whole-genome sequence, a large number of SSRs and SNPs have recently been detected in pulse crops (Table 1). For example, massive-scale SSR markers including 2,393 and 28,503 SSRs were developed in pea and faba bean, respectively, using Roche 454-FLX sequencing (Kaur et al. 2011; Yang et al. 2012). Likewise, thousands of SNP markers were identified in pea (50,000) and lentil (44,879) using NGS technologies such as Roche 454-FLX and Illumina Genome Analyzer (GA) (Sharpe et al. 2013; Sindhu et al. 2013).

**Table 1:** List of available genomic tools in selected pulse crops

Genomic Resources	Cowpea	Pea	Lentil	Faba bean
Mapping resources				
Traditional bi-parental populations	~30 (including Sesquipedalis group) (Lucas et al. 2011; Muchero et al. 2009a, b; Ouedraogo et al. 2001, 2001, 2012)	~25 (McPhee 2007; Rubiales et al. 2011)	~20 (Ford et al. 2007; Pérez de la Vega et al. 2011)	~ 20 (Arbaoui et al. 2008; Ma et al. 2013; Torres et al. 2006)
Second-generation populations like MAGIC/NAM	In progress	–	–	–
Reverse genetics resources				
TILLING population	–	Two sets comprising 3,027 and 4,704 lines (Dalmais et al. 2008; Triques et al. 2007)	–	–
BAC-tools				
BAC libraries	3 (Yu 2012)	2 (Yu, 2012)	–	–
BESs	30,527 (Barrera-Figueroa et al. 2011)	–	–	–
Physical maps	10 × coverage (Close et al. 2011)	–	–	–
Genetic markers				
Genomic SSRs				
Enriched library based	44 (Li et al. 2001)	434 (Loridon et al. 2005)	360 (Andeden et al. 2013), ~75 SSRs (Durán et al. 2004; Hamwieh et al. 2005, 2009)	73 (Zeid et al. 2009)
Gene space read (GSR)/BES and NGS based	1,071 (Gupta and Gopalakrishna 2010); 712 (Andargie et al. 2011); 1, 372 (Xu et al. 2010, 2011a, b)	43 (Burstin et al. 2001)	–	28,503 (Yang et al. 2012)
EST-SSRs	410 (Xu et al. 2010)	80 (De Caire et al. 2011); 2,397 (Kaur et al. 2012)	2,393 (Kaur et al. 2011); 5,673 (Verma et al. 2013)	802 (Kaur et al. 2012); 336 (Kaur et al. 2014)
SNPs	1,536 (Lucas et al. 2011; Muchero et al. 2009a; Xu et al. 2011a, b)	63 (Aubert et al. 2006a, 2006b); 384 (Deulvot et al. 2010); 36,188 (Leonforte et al. 2013); 35,455 (Duarte et al. 2014)	44,879 (Sharpe et al. 2013); 1,095 (Temel et al. 2014)	75 (Cottage et al. 2012); 14,522 (Kaur et al. 2014)
Transcriptomic resources				
ESTs deposited at NCBI http://www.ncbi.nlm.nih.gov/dbEST/dbEST_summary.html (dbEST release 1st Jan 2013)	1,87,487	1,85,76	9,513	5,510
Transcriptome assemblies	1 (Muchero et al. 2009a)	3 (Duarte et al. 2014; Franssen et al. 2011; Kaur et al. 2012)	3 (Kaur et al. 2011; Sharpe et al. 2013; Verma et al. 2013)	1 (Kaur et al. 2012)
Genetic linkage maps				
Population specific	~25 (Lucas et al. 2011; Muchero et al. 2009a, b; Ouedraogo et al. 2001, 2002, 2012; Timko et al. 2007)	~35 (McPhee 2007; Rubiales et al. 2011)	~20 (Andeden et al. 2013; Ford et al. 2007; Pérez de la Vega et al. 2011)	~10 (Gutiérrez et al. 2013; Ma et al. 2013; Torres et al. 2011)
Consensus/composite	2 (Muchero et al. 2009a; Lucas et al. 2011)	7 (Aubert et al. 2006a, b; Bordat et al. 2011; Duarte et al. 2014; Hamon et al. 2011, 2013; Loridon et al. 2005; Weeden et al. 1998)	–	4 (Román et al. 2004; Satovic et al. 1996, 2013; Vaz Patto et al. 1999)
Whole-genome sequence	In progress	In progress	In progress	–

Interestingly, the discovery of high-density SNP markers is complemented with the establishment of ultra HTP genotyping assays like Illumina GoldenGate (GG) and Infinium assays, which are able to accommodate up to 3,000 and 4 million SNPs, respectively (Deschamps et al. 2012). Informative SNPs were chosen for designing robust GG assays and as a result 768-/1,536-SNPs based GG platforms have become available in cowpea (Lucas et al. 2011; Muchero et al. 2009a, 2013), pea (Duarte et al. 2014; Leonforte et al. 2013; Sindhu et al. 2013), lentil (Kaur et al. 2013; Sharpe et al. 2013) and faba bean (Kaur et al. 2014). Further, increasing number of re-sequencing database in coming days will allow identification of more SNPs and, consequently, HTP cost-effective genotyping assays using only informative SNPs will become available in all pulse crops.

Due to major shortcomings of GG and Infinium assays including cost-prohibitive designing and low flexibility, some customized SNP detection systems like competitive allele-specific PCR (KASPar) have been introduced to incorporate small to moderate number of SNPs for specific applications (Hiremath et al. 2012; Khera et al. 2013; Kumar et al. 2012; Saxena et al. 2012). Given the flexibility mentioned above, the KASPar assay was used for typing SNPs in asparagus bean (Xu et al. 2012a), lentil (Fedoruk et al. 2013; Sharpe et al. 2013) and faba bean (Cottage et al. 2012). Similarly, another custom-designed Illumina Veracode assay was employed for genotyping a set of 384 SNP markers in pea (Deulvot et al. 2010). Utilization of such automated genotyping systems not only enhances the speed of genotyping, but also ensures better accuracies in SNP typing. Apart from SNPs, diversity arrays technology (DArT) is another second-generation automated platform that enables genotyping of hundreds to thousands of genome-wide DNA markers with great precision. Successful implementation of DArT system has been reported in several pulse crops including chickpea and common bean for genetic linkage mapping and genetic diversity estimation (Briñez et al. 2012; Thudi et al. 2011). However, among the pulse crops presented here, to our knowledge DArT markers have not been applied so far.

### Transcriptome and genome assemblies

Transcriptome assemblies are excellent genomic resources to capture the gene space for both basic and applied studies. Transcriptome assemblies facilitate detailed comparative analyses across different genera and discovery of functionally relevant markers (FMs), especially EST-SSR, SNP, intron-targeted primer (ITP) or intron spanning region (ISR) markers (Agarwal et al. 2012; Kudapa et al. 2012). More importantly, in case of crops like pea, lentil and faba bean with large and poorly characterized genomes, comprehensive transcriptome assemblies offer a means to directly access the gene space and causative functional polymorphisms, thus yielding valuable insights about the genome organization.

Initially, Sanger sequencing of c-DNA libraries generated transcriptomics resources such as ESTs for various crop species. For instance, a total of 183,118 ESTs were recovered through sequencing of nine c-DNA libraries in cowpea (Muchero et al. 2009a). Recently, transcriptome/cDNA library sequencing using 454 GS-FLX Titanium (generating longer reads) and Illumina GA/GAIIx systems (comparatively shorter reads) has appeared as a potential alternative to leverage the genomic resource repertoire. Deep transcriptome sequencing has been performed in pea (Duarte et al. 2014; Franssen et al. 2011; Kaur et al. 2012), lentil (Sharpe et al. 2013; Verma et al. 2013) and faba bean (Kaur et al. 2012). As a result of this HTP sequencing, massive transcriptomic data were obtained in the form of high-quality sequence reads in the selected pulse crops, viz. pea (720,324 reads), lentil (847,824 reads) and faba bean (304,680), and the transcriptome assemblies consisted of 70,682, 84,074 and 60,440 unigenes, respectively.

Based on the different approaches chosen for assembly of NGS reads, various kinds of transcriptome assemblies, viz. *de novo*, reference based and hybrid are being established in these pulse crops (Agarwal et al. 2012; Kudapa et al. 2012). The immense potential of NGS was also explored for whole-genome transcript profiling in faba bean, and NGS in combination with super serial analysis of gene expression (SAGE) led to the generation of 1,313,009 tags shedding new light on the transcriptional changes that take place during faba bean–*Ascochyta fabae* interaction (Madrid et al. 2013). Moreover, from functional genomics concerns, faba bean is particularly important as it has served as an excellent system for understanding the kinetics of stomatal movements in plants (Chen et al. 2004; Dietrich et al. 2001; Gao et al. 2005; Hanstein and Felle 2002). In addition to transcriptome, low-depth 454 sequencing was successfully utilized to uncover the repetitive DNA in the pea genome, which enabled a genome-wide characterization of the major repeat families and comparison of repeat composition with other legume species including soybean and *Medicago* (Macas et al. 2007).

On account of their shorter sequence reads and higher error rates (as compared to Sanger sequencing), NGS methods were initially considered suitable for re-sequencing of genotypes where a high-quality reference genome sequence was available (Imelfort and Edwards 2009; Varshney et al. 2009b). With continuous refinements being made in computational algorithms that are used for assembly and alignment, NGS was also applied to *de novo* whole-genome sequencing especially in the crops with moderate-sized genomes and even in the absence of physical maps (Varshney et al. 2011). In contrast to the BAC by BAC method, which is very tedious involving construction of BAC libraries, sequencing of BACs, development of a physical map and the determination of MTP, the current de novo genome assembly using whole-genome shotgun (WGS) approach is straightforward, cost-effective and time saving (Imelfort and Edwards 2009; Venter et al. 1996).

In addition to model legume species like *Medicago truncatula* (Young et al. 2011), *Lotus japonicus* (http://www.kazusa.or.jp/lotus/index.html), whole/draft genome sequence has become available for soybean (Schmutz et al. 2010), pigeonpea (Varshney et al. 2011) and chickpea (Varshney et al. 2013b). More recently, 52 % (598 Mb) genome has been assembled for lupin (Yang et al. 2013). Among pulses selected for discussion here, assembling the gene space in cowpea is underway (Tim Close, personal communication). Similarly, efforts have been initiated to sequence genomes of pea and lentil. In case of lentil, a draft (23×) of the genome assembly has recently been generated for the reference genotype ‘CDC Redberry’ (Ramsay et al. 2014). The complexity and large genome size coupled with small research community have not allowed undertaking genome sequencing of faba bean.

NGS methods are also being employed for whole-genome re-sequencing (WGRS) and restriction site-associated DNA (RAD) sequencing of germplasm lines for exploring genetic diversity and population dynamics (Varshney et al. 2013b). Like the above-mentioned techniques, genotyping by sequencing (GBS) is another NGS-based platform that allows simultaneous discovery and mapping of several thousands of genetic markers (Davey et al. 2011). In lentil, the NGS-GBS approach has facilitated detection and mapping of genome-wide SNPs (Temel et al. 2014). Advances in sequencing technologies and collaborative efforts are expected to deliver draft genome sequences in all the pulse crops in the very recent future. It is also anticipated that re-sequencing of germplasm collections in these pulse crops will provide estimates on genome diversity and detailed population structure of germplasm collections.

## Trait mapping/gene(s) discovery in pulse crops

Identification of a gene/QTL underlying the trait of interest is the most critical step while proceeding for marker-assisted selection (MAS)/GAB. Among various genomic resources, molecular markers are of direct application in crop breeding, as these are heavily deployed in trait mapping studies using either family-based linkage (FBL) mapping approaches or germplasm-based association mapping (AM) (Mackay and Powell 2007). An appropriately built experimental population with considerable size lies at the core of FBL-based QTL discovery studies (Mitchell-Olds 2010). Alternatively, non-experimental population or a set of genetically diverse genotypes can be used for uncovering the genetic architecture of important traits via linkage disequilibrium (LD) analysis or AM (Mackay and Powell 2007). Trait mapping using linkage or association analysis corresponds to a forward genetics approach, in which phenotypic expression is usually known and the phenotypic variation is therefore targeted for detecting causal genetic polymorphisms. In contrast, a reverse genetics method, more precisely a *locus*-*to*-*phenotype* approach, relies on determination of the function of a known sequence (McCallum et al. 2000).

### Genetic populations: bi-parental and multi-parental mapping resources

The family-based populations are usually derived from two genotypes showing sufficient phenotypic diversity for few traits. Among the different types of populations available, the genetic constitution of F_2_ or backcross (BC) harbours considerable heterozygosity, thus limiting opportunities for replicated measurements (Collard et al. 2005). By contrast, the nearly homozygous nature of recombinant inbred (RI) populations enables multi-location and multi-season screening of the population, which eventually enhances the strength of QTL detection (Varshney et al. 2009c). In pulses, numerous experimental populations have been developed belonging to both narrow (intraspecific)- and broad (interspecific)-based crosses, facilitating construction of several population-specific genetic maps and molecular tagging/mapping of the targeted traits (Table 2; Table 3a, b).

**Table 2:** Detailed list of genetic linkage maps in the four major pulse crops [genetic maps with moderate to high marker density (≥100 loci) are included]

Name of population	Type of population	Population size	Number of loci	Map length (cM)	Types of markers	References
Cowpea						
524 B × IT84S 2049	RIL	94	181	972	RFLP, RAPD, AFLP, biochemical and morphological	Menéndez et al. (1997)
524 B × IT84S 2049	RIL	94	440	2670	RFLP, RAPD, AFLP, RGA, biochemical and morphological	Ouedraogo et al. (2001)
Sanzi × Vita 7	RIL	92	139	1,620	AFLP and SSR	Omo-Ikerodah et al. (2008)
CB 46 × IT93 K 503-1	RIL	103	388	601	SNP	Muchero et al. (2009a, 2011)
524 B × IT84S 2049	RIL	79	436	665	SNP	Muchero et al. (2009a)
Dan Ila × TVu 7778	RIL	109	288	665	SNP	Muchero et al. (2009a)
Yacine × 58-77	RIL	114	415	657	SNP	Muchero et al. (2009a)
TVu14676 × IT84S 2246-4	RIL	137	349	600	SNP	Muchero et al. (2009a)
CB27 × 24-125 B-1	RIL	90	299	651	SNP	Muchero et al. (2009a)
IT93 K 503–1 × CB46	RIL	127	306	643	AFLP	Muchero et al. (2009b, 2011)
DanIla × TVu7778	RIL	113	282	633	SNP	Agbicodo et al. (2010)
524 B × 219-01	RIL	159	202	677	SSR	Andargie et al. (2011)
CB 27 × IT97 K 566-6	RIL	95	438	505	SNP	Lucas et al. (2011)
CB 27 × IT82E 18	RIL	166	430	701	SNP	Lucas et al. (2011)
CB 27 × UCR 779	RIL	58	560	489	SNP	Lucas et al. (2011)
IT84S 2246 × IT93 K 503	RIL	130	374	639	SNP	Lucas et al. (2011)
IT84S 2246 × Mouride	RIL	92	347	595	SNP	Lucas et al. (2011)
LB30#1 × LB1162 #7	RIL	95	180	409	SNP	Lucas et al. (2011)
ZN016 × Zhijiang282	RIL	114	375	745	SSR and SNP	Xu et al. (2011a)
(JP81610 × JP89083) × JP81610	BC_1_F_1_	190	226	852	SSR	Kongjaimun et al. (2012a, b, 2013)
JP81610 × JP89083	F_2_	188	113	977	SSR	Kongjaimun et al. (2012b, 2013)
524B × 219-01	RIL	159	206	677	SSR and morphological marker	Andargie et al. (2013, 2014)
Pea						
Primo × OSU442-15	F_2_	102	207	1,330	RFLP, RAPD and AFLP	Gilpin et al. (1997)
JI 15 JI 399	RIL	–	179	1,400	–	Hall et al. (1997)
JI 281 × I 399	RIL	–	318	2,300	–	Hall et al. (1997)
Térèse × K 586	RIL	139	240	1,139	RFLP, RAPD, morphological and others	Laucou et al. (1998)
JI 281 × JI 399	RIL	–	355	1,881	RFLP, RAPD, morphological and others	Laucou et al. (1998)
Primo × OSU442-15	F_2_	102	199	1,510	RFLP, RAPD and AFLP	McCallum et al. (1997)
JI 1794 × Slow	RIL	51	235	1,289	RFLP, RAPD, AFLP, isozyme and morphological	Timmerman-Vaughan et al. (1996)
Puget × 90-2079	RIL	127	324	1,094	AFLP, RAPD, SSR, ISSR, STS, isozyme and morphological	Pilet-Nayel et al. (2002)
JI 15 × JI 399	F_2_	120	137	710	SSAP	Knox and Ellis (2002)
JI 15 × JI 399	RIL	89	137	565	SSAP	Knox and Ellis (2002)
Wt 10245 × Wt 11238	F_2_	114	204	2,416	RAPD, AFLP, ISSR, STS, CAPS, isozyme and morphological	Irzykowska and Wolko (2004)
Carneval × MP 1401	RIL	88	207	1,274	AFLP, RAPD and STS	Tar’an et al. (2003b, 2004)
DP × JI 296	RIL	135	206	1,061	RAPD, SSR, STS and morphological	Prioul et al. (2004)
Champagne × Térèse	RIL	164	189	–	SSR, RAPD and morphological	Loridon et al. (2005)
Shawnee × Bohatyr	RIL	187	302	–	SSR, RAPD, isozyme and morphological	Loridon et al. (2005); McPhee et al. (2012)
Primo × OSU442-15	F_2_	227	108	1,369	RFLP, RAPD, AFLP and STS	Timmerman-Vaughan et al. (2005)
JI 281 × JI 399	RIL	71	153		RFLP and morphological	Ellis et al. (1992); (McPhee 2007)
Orb × CDC Striker	RIL	90	224	900	SSR and AFLP	Ubayasena et al. (2010)
P 665 × Messire P 665	RIL	111	246	1,214	RAPD, STS, EST, isozyme and morphological	Fondevilla et al. (2008, 2010)
Cameor × Ballet	RIL	207	152	1,140	–	Bourion et al. (2010)
DSP × 90-2131	RIL	111	168	1,046	RAPD, SSR, genic and morphological marker	Hamon et al. (2013)
Orb × CDC Striker	RIL	–	255	479	SNP	Sindhu et al. (2013)
Pennant × ATC113	F_2_	188	155	2,686	SSR	Aryamanesh et al. (2014)
Kaspa × Parafield	RIL	134	458	1,916	SSR and SNP	Leonforte et al. (2013)
Lentil						
*L. culinaris* ssp. *orientalis* × *L. culinaris*	RIL	86	177	1,073	RAPD, AFLP, RFLP and morphological	Eujayl et al. (1998)
ILL5588 × ILL7537	F_2_	150	114	784	RAPD, ISSR and RGA	Rubeena et al. (2003)
ILL 5588 × L 692-16-1(s)	RIL	86	283	751	SSR and AFLP	Hamwieh et al. (2005)
Lupa × Boiss	F_2_	113	161	2,172	RAPD, ISSR, AFLP, SSR and morphological	Durán et al. (2004); Fratini et al. (2007)
Eston × PI 320937	RIL	94	207	1,868	AFLP, RAPD and SSR	Tullu et al. (2006, 2008)
Precoz × WA 8649041	RIL	94	166	1,396	AFLP, ISSR, RAPD and morphological	Tanyolac et al. (2010)
ILL 6002 × ILL 5888	RIL	206	139	1,565	SSR, RAPD, SRAP and morphological	Saha et al. (2010, 2013)
WA 8649090 × Precoz	RIL	106	130	1,192	RAPD, ISSR and AFLP	Kahraman et al. (2004, 2010)
L 830 × ILWL 77	F_2_	114	199	3,843	RAPD, ISSR and SSR	Gupta et al. (2012b)
Digger (ILL 5722) × NorthWeld (ILL 5588)	RIL	94	211	1,392	ISSR, RAPD, ITAP and SSR	Gupta et al. (2012c)
CDC Robin × 964a-46	RIL	139	543	835	SSR and SNP	Sharpe et al. (2013)
*L. culinaris* ssp. *orientalis* × *L. culinaris*	F_2_	113	190	2,234	RAPD, SRAP, SSR, CAPS and presence–absence polymorphism	de la Puente et al. (2013)
CDC Robin × 964a-46	RIL	139	577	697	SNP, SSR and seed colour loci	Fedoruk et al. (2013)
Cassab × ILL2024	RIL	126	318	1,178	SSR and SNP	Kaur et al. (2013)
PI 320937 × Eston	RIL	96	194	840	AFLP, SSR and SNP	Sever et al. (2014)
Precoz × WA 8649041	RIL	101	519	540	SNP	Temel et al. (2014)
ILL 8006–BM (Barimasur-4) × CDC Milestone	RIL	–	149	497	AFLP, SSR and SNP	Aldemir et al. (2014)
Faba bean						
Vf 6 × Vf 136	F_2_	196	121	1,445	RAPD, isozyme and seed protein	Román et al. (2002)
29 H × Vf 136	F_2_	159	103	1,308	RAPD, SSR, isozymes and seed protein genes	Avila et al. (2005)
Vf 6 × Vf 27	RIL	94	127	1,686	ITAP	Ellwood et al. (2008)
Cote d’Or 1 × BPL 4628	RIL	101	132	1,635	RAPD and morphological markers	Arbaoui et al. (2008)
Vf 6 × Vf 136	RIL	165	277	2,857	RAPD, EST, SCAR, SSR, STS, ISP and isozymes	Díaz-Ruiz et al. (2009a)
Vf 6 × Vf 27	RIL	124	258	1,875	RAPD, SSR, isozymes, seed proteins, morphological and EST-derived markers	Cruz-Izquierdo et al. (2012)
29 H × Vf 136	RIL	119	172	1,402	RAPD, SSR, RGA, seed storage protein, DR (defence-related) gene and EST-derived markers	Gutiérrez et al. (2013)
91825 × K 1563	F_2_	129	128	1,587	SSR	Ma et al. (2013)
Icarus × Ascot	RIL	95	522	1,217	SSR and SNP	Kaur et al. (2014)

**Table 3:** Trait mapping in selected pulse crops

Trait	Name of the population	Associated marker(s)	Reference
a) BSA-based molecular tagging			
Cowpea			
Cowpea golden mosaic virus	IT97 K-499-35 × Canapu T16	AFLP	Rodrigues et al. (2012)
*Striga* resistance	Tvx 3236 × IT82D-849	AFLP	Ouedraogo et al. (2001)
	Tvu 14676 × IT84S-2246–4	AFLP	Ouedraogo et al. (2001)
	IT84S-2246 × Tvu14676	SCAR	Ouedraogo et al. (2012)
	IT93 K-693-2 × IAR1696	AFLP/SCAR	Boukar et al. (2004)
Pea			
Development funiculus (def)	DGV × PF	AFLP/STS	von Stackelberg et al. (2003)
Determinate growth (det)	JI2121 × Térèse	RAPD	Rameau et al. (1998)
Fascinated stem (fa)	JI814 × Térèse	RAPD	Rameau et al. (1998)
Increased branching (rms)	K524 × Térèse	RAPD	Rameau et al. (1998)
	WL6042 × Térèse	RAPD	Rameau et al. (1998)
	M3T-946 × Torsdag	RAPD	Rameau et al. (1998)
Nodulation loci	P56 × JI15 P2 × JI281 P54 × JI281	RFLP	Schneider et al. (2002)
Pea seed-borne mosaic virus (PSbMV)	88V1.11 × 425	RFLP	Timmerman et al. (1993)
Photoperiod insensitivity (dne)	K218 × Térèse	RAPD	Rameau et al. (1998)
Photoperiod insensitivity (sn)	HL59 × Térèse	RAPD	Rameau et al. (1998)
Powdery mildew	Radley × Highlight	RAPD/SCAR	Tiwari et al. (1998)
	Majoret × 955180	SSR	Ek et al. (2005)
	Solara × Frilene-derived mutant	SCAR	Pereira et al. (2010)
	Sparkle × Mexique	RAPD/SCAR	Tonguç and Weeden (2010)
*Fusarium* wilt (race 1) resistance	Green Arrow × PI 179449	TRAP	Kwon et al. (2013)
Lentil			
Ascochyta blight resistance	ILL5588 × ILL6002	RAPD	Ford et al. (1999)
	Eston × Indian head	RAPD/SCAR	Chowdhury et al. (2001)
Fusarium vascular wilt	ILL5588 × L692–16-l (s)	RAPD	Eujayl et al. (1998)
Radiation frost tolerance (*Frt*)	ILL5588 × L692–16-l (s)	RAPD	Eujayl et al. (1999)
Anthracnose resistance (LCt-2)	Eston × PI 320937	AFLP/RAPD	Tullu et al. (2003)
Faba bean			
Rust resistance	2N52 × VF-176	RAPD	Avila et al. (2003)
Determinate growth habit	Verde Bonita × 2N52	CAPS	Avila et al. (2006)
Reduced vicine and convicine content	Vf 6 × 1268	CAPS	Gutiérrez et al. (2006)
Absence of tannin	Vf 6 × zt-1 line	SCAR	Gutiérrez et al. (2007)
	Vf 6 × zt-2 line	SCAR	Gutiérrez et al. (2008)

Trait	Name of population	Marker associated with QTL(s)	PV explained by the QTLs (%)^*^	Reference
b) Linkage map/QTL-based molecular mapping				
Cowpea				
Cowpea bacterial blight (CoBB) resistance	DanIla × TVu7778	SNP	22	Agbicodo et al. (2010)
Drought-induced senescence	IT93K503–1 × CB46	AFLP	24	Muchero et al. (2009b)
Flower bud thrips resistance	Sanzi × Vita 7	AFLP	77	Omo-Ikerodah et al. (2008)
Foliar thrips	CB46 × IT93 K-503-1 and CB27 × IT82E − 18	SNP	32	Lucas et al. (2012)
Hastate leaf shape	Sanzi × Vita 7	SNP	74	Pottorff et al. (2012a)
Pod fibre layer thickness	524B × 219-01	SSR	17	Andargie et al. (2011)
Pod length	(JP81610 × JP89083) × JP81610	SSR	31	Kongjaimun et al. (2012a)
Domestication-related traits	(JP81610 × JP89083) × JP81610	SSR	53	Kongjaimun et al. (2012b)
Seed weight	IT2246-4 × TVNI 963	RFLP	53	Fatokun et al. (1992)
	524B × 219-01	SSR	19	Andargie et al. (2011)
Charcoal rot resistance	IT93 K-503-1 × CB46	SNP and AFLP	40	Muchero et al. (2011)
Flower and seed coat colour	ZN016 × Zhijiang 28-2	SNP	–	Xu et al. (2011b)
Time of flower opening	524 B × 219-01	SSR	30	Andargie et al. (2013)
Days to flower	524 B × 219-01	SSR	19	Andargie et al. (2013)
	ZN016 × ZJ282	SNP	32	Xu et al. (2013)
Nodes to first flower	ZN016 × ZJ282	SNP	22	Xu et al. (2013)
Pod number per plant	ZN016 × ZJ282	SSR	20	Xu et al. (2013)
Leaf senescence	ZN016 × ZJ282	SNP	29	Xu et al. (2013)
Floral scent compounds	524 B × 219-01	SSR	60	Andargie et al. (2014)
Heat tolerance	CB27 × IT82E − 18	SNP	18	Lucas et al. (2013a)
Seed size	Eight different populations	SNP	47	Lucas et al. (2013b)
*Fusarium* wilt resistance (Fot race 3)	CB27 × 24-125B-1	SNP	28	Pottorff et al. (2012b)
*Fusarium* wilt resistance (Fot race 4)	IT93 K-503-1 9 CB46	SNP	47	Pottorff et al. (2014)
	CB27 × 24-125B-1	SNP	40	Pottorff et al. (2014)
	CB27 × IT82E − 18	SNP	27	Pottorff et al. (2014)
Pod tenderness	(JP81610 × JP89083) × JP81610	SSR	50	Kongjaimun et al. (2013)
	JP81610 × JP89083	SSR	43	Kongjaimun et al. (2013)
Pea				
*Aphanomyces* root rot	Puget × 90-2079	AFLP	47	Pilet-Nayel et al. (2002)
	Baccara × PI 180693	–	49	Hamon et al. (2011)
	Baccara × 552	–	21	Hamon et al. (2011)
	DSP × 90-2131	–	60	Hamon et al. (2013)
*Ascochyta* blight resistance	A88 × Rovar	–	35	Timmerman-Vaughan et al. (2002)
	DP × JI296	–	74	Prioul et al. (2004)
	P665 × Messire. P665	–	75	Fondevilla et al. (2008)
Days to maturity	Carneval × MP1401	–	34	Tar’an et al. (2004)
Frost resistance	Champagne × Terese	–	45	Dumont et al. (2009)
Grain yield	Carneval × MP1401	–	38	Tar’an et al. (2004)
Lodging resistance	Carneval × MP1401	AFLP/SCAR	58	Tar’an et al. (2003b, 2004)
*Mycosphaerella* blight resistance	Carneval × MP1401	–	36	Tar’an et al. (2003b, 2004)
Plant height	Erygel × 661	RFLP	19	Dirlewanger et al. (1994)
	Carneval × MP1401	–	65	Tar’an et al. (2003b, 2004)
Plant maturity	A26 × Rovar	–	27	Timmerman-Vaughan et al. (2004)
Seed protein concentration	Carneval × MP1401	–	45	Tar’an et al. (2004)
Seed weight	Primo × OSU442-15	RAPD	62	Timmerman-Vaughan et al. (1996)
Yield component and developmental traits	Primo × OSU442-15	–	62	Timmerman-Vaughan et al. (2005)
Yield-related traits and seed protein content	Wt10245 × Wt11238	–	56	Irzykowska and Wolko (2004)
Pea weevil	Pennant × ATC113	SSR	43	Aryamanesh et al. (2014)
*Fusarium* wilt (race 2) resistance	Shawnee × Bohatyr	SSR	80	McPhee et al. (2012)
Salt tolerance	Kaspa × Parafield	SNP	19	Leonforte et al. (2013)
Lentil				
*Ascochyta* blight resistance	ILL 7537 × ILL 6002	AFLP	47	Rubeena et al. (2003)
	Eston × PI320937	AFLP and RAPD	50	Tullu et al. (2006)
	Digger (ILL5722) × NorthWeld (ILL5588)	ITAP, SSR and ISSR	61	Gupta et al. (2012b)
	ILL5588 × ILL7537 and ILL7537 × ILL6002	–	50	Rubeena et al. (2006)
Earliness	Eston × PI320937	RAPD and AFLP	46	Tullu et al. (2008)
Plant height	Eston × PI320937	AFLP and SSR	40	Tullu et al. (2008)
	Lupa × Boiss	–	38	Fratini et al. (2007)
*Stemphylium* blight resistance	ILL-6002 × ILL-5888	SRAP and RAPD	46	Saha et al. (2010)
Winter hardiness	WA8649090 × Precoz	ISSR	43	Kahraman et al. (2010)
Seed thickness	CDC Robin × 964a-46	Morphological marker (cotyledon colour locus (Yc))	38	Fedoruk et al. (2013)
Seed plumpness	CDC Robin × 964a-46	Cotyledon colour locus (Yc)	40	Fedoruk et al. (2013)
Days to 50 % flowering	CDC Robin × 964a-46	Cotyledon colour locus (Yc)	35	Fedoruk et al. (2013)
	ILL 6002 × ILL 5888	SSR/RAPD/SRAP	20	Saha et al. (2013)
Seed diameter	Lupa × Boiss	–	37	Fratini et al. (2007)
	ILL 6002 × ILL 5888	SSR/RAPD/SRAP	32	Saha et al. (2013)
Seed weight	Lupa × Boiss	–	18	Fratini et al. (2007)
	ILL 6002 × ILL 5888	SSR/RAPD/SRAP	18	Saha et al. (2013)
Boron tolerance	Cassab × ILL2024	SNP	71	Kaur et al. (2013)
Faba bean				
*Ascochyta* blight resistance	29 H × Vf 136	RAPD	45	Avila et al. (2004)
	Vf 6 × Vf 136	RAPD	25	Román et al. (2003)
	Vf 6 × Vf 136	RAPD	24	Díaz-Ruiz et al. (2009a)
	Icarus × Ascot	SNP	20	Kaur et al. (2014)
Broomrape resistance	Vf 6 × Vf 136	RAPD	35	Román et al. (2002)
	Vf 6 × Vf 136	RAPD	43	Díaz-Ruiz et al. (2009b)
	29 H × Vf 136	RAPD	33	Gutiérrez et al. (2013)
Floral characters	29 H × Vf 136	RAPD	20	Avila et al. (2005)
Days to flowering	Vf 6 × Vf 27	SSR	28	Cruz-Izquierdo et al. (2012)
Flowering length	Vf 6 × Vf 27	EST-derived marker	31	Cruz-Izquierdo et al. (2012)
Pod length	Vf 6 × Vf 27	SSR	25	Cruz-Izquierdo et al. (2012)
Number of ovules per pod	Vf 6 × Vf 27	EST-derived marker	27	Cruz-Izquierdo et al. (2012)
Number of seeds per pod	Vf 6 × Vf 27	RAPD	26	Cruz-Izquierdo et al. (2012)
Seed weight	–	RAPD	30	Vaz Patto et al. (1999)
Yield characters	29 H × Vf 136	RAPD	58	Avila et al. (2005)
Frost tolerance	Coted’Or 1 × BPL 4628	RAPD	40	Arbaoui et al. (2008)
Fatty acid content	Coted’Or 1 × BPL 4628	RAPD	63	Arbaoui et al. (2008)

* QTLs with the highest phenotypic variation (PV) are shown and only major effect QTLs with PV ≥ 10 % are considered

Bi-parental mapping populations are endowed with greater *power* for detection of QTLs; however, the mapping resolution i.e. *precision* is not adequate, thus making these populations (except NILs) suitable for *coarse* mapping only (Cavanagh et al. 2008). The map resolution can be enhanced by (1) incorporating multiple alleles in a segregating population and (2) introducing provisions for inter-mating in the advanced generations (Korte and Farlow 2013). In view of the above considerations, a novel methodology known as multi-parent advanced generation inter-cross (MAGIC) has been introduced in plants (Mackay and Powell 2007). The MAGIC scheme is capable of exploiting wide genetic variation existing among the multiple founders (Cavanagh et al. 2008). Further, provisions for inter-mating open up new opportunities for recovery of a large number of informative recombinants, which is otherwise not feasible in case of traditional bi-parent populations.

Like RI populations, MAGIC lines represent immortal mapping resource suitable for *joint linkage* *association* analysis (Xu et al. 2012b). Recent achievements of MAGIC in *Arabidopsis*, wheat and rice (see Bandillo et al. 2013) have placed emphasis towards inclusion of multiple parents while generating experimental populations in pulse crops. Consequently, with support of the CGIAR Generation Challenge Programme (GCP), development of meta-population derived from eight founders (or MAGIC, with 8 parental lines) is underway in cowpea (Ribaut et al. 2012; https://sites.google.com/site/ijmackay/work/magic). Besides fine mapping of QTL(s), the stable MAGIC lines have direct or indirect applications in germplasm enhancement and cultivar development (Bandillo et al. 2013). Likewise, another multi-parent based approach, i.e. nested association mapping (NAM) also permits both FBL and LD analyses (Cook et al. 2012; McMullen et al. 2009; Tian et al. 2011). The availability of genome sequence of the reference genotype in almost all the major pulse crops will help greatly for using the reference genotype as *common* parent for developing a series of *connected* bi-parental RI populations that constitutes the NAM design (McMullen et al. 2009).

### Genetic linkage maps and QTLs

Recent advances in marker systems starting from limited morphological markers to abundant sequence-based markers have taken genetic mapping to the next level where the mapping populations can be explored best for superior alleles. In the context of genetic mapping, pea is one of the pioneer crops in which several morphological markers were successfully mapped using classical genetics approaches. For instance, the pea mutation map was developed by mapping 169 morphological markers (Blixt 1972). Similar instances were reported in other pulse crops like lentil, where the initial genetic maps were based on morphological and isozyme markers (Zamir and Ladizinsky 1984).

Highly saturated genetic maps and precisely mapped QTLs are the essential tools for undertaking GAB. A quantum leap in the marker systems towards easy-to-use SNP markers has led to the development of highly saturated genetic maps in the major pulse crops. The core mapping populations were used to develop functional or transcript maps in these crops such as SNP-based maps developed for ‘China × Cameor’ and ‘Orb × CDC Striker’ in pea (Deulvot et al. 2010; Sindhu et al. 2013), ‘CDC Robin × 964a-46’ (LR-18) in lentil (Fedoruk et al. 2013; Sharpe et al. 2013) and ‘Icarus × Ascot’ in faba bean (Kaur et al. 2014). These genetic maps provided map locations to a number of markers with considerable genome coverage, e.g. 543 loci (834.7 cM) in lentil (Sharpe et al. 2013). Further, a detailed list of population-specific genetic maps in four selected pulse crops is presented in Table 2.

In parallel, the segregation data from diverse mapping populations are analysed to synthesize a much broader and species-specific genetic map known as ‘consensus’ or ‘composite’ map (see Bohra 2013). Moderate- to high-density consensus maps have been reported in pea (Hamon et al. 2011, 2013; Loridon et al. 2005), cowpea (Lucas et al. 2011; Muchero et al. 2009a) and faba bean (Román et al. 2004; Satovic et al. 1996, 2013; Vaz Patto et al. 1999) offering higher mapping resolution and better genome coverage. Among pulse crops, a comprehensive consensus map was established for cowpea using ~700 individuals belonging to six different RILs. The six component or population-specific genetic maps had loci ranging from 288 to 436 with several common SNPs mapped in different populations. Subsequently, with the help of *bridge* SNPs, all six component maps were combined into a single, high-density and robust consensus map with 645 bins encompassing 928 loci and 680 cM (Muchero et al. 2009a). This map was further refined by Lucas et al. (2011) with 1,107 SNPs arranged in 856 bins, thus increasing marker density from 0.73 cM (Muchero et al. 2009a) to 0.61 cM (http://harvest.ucr.edu). Similarly, notable consensus maps were developed for pea and faba bean comprising 619 loci (1,513 cM) and 729 loci (4,602 cM), respectively (Hamon et al. 2013; Satovic et al. 2013). More recently, Duarte et al. (2014) combined data from four different RILs in pea and synthesized a highly saturated consensus genetic map with 2,070 loci covering 1,255 cM. Moreover, the meta-QTL analysis using consensus/composite maps enable placing of several QTLs from multiple populations onto a single genetic map, thus enhancing the QTL resolution and additionally incorporating more informative markers into the QTL-containing regions (Hamon et al. 2013).

The linkage map-based QTLs controlling several agriculturally important traits have been identified in almost all the major pulse crops (Table 3). In the absence of a genetic linkage map, bulked segregants analysis (BSA) is usually performed to find DNA markers tightly associated with the concerned trait, mostly resistance to biotic stresses (Table 3). BSA using NILs is a powerful mapping strategy widely used for understanding marker–trait relationships (Gepts et al. 2008). The noteworthy examples of BSA-based molecular tagging in pulses include various types of markers such as random amplification of polymorphic DNA (RAPD)/amplified fragment length polymorphism (AFLP)/sequence-characterized amplified region (SCAR)/cleaved amplified polymorphic sequence (CAPS) markers, which were employed for screening *Ascochyta* blight resistance in lentil (Chowdhury et al. 2001), *Striga* resistance in cowpea (Boukar et al. 2004; Ouedraogo et al. 2001), powdery mildew in pea (Pereira et al. 2010) and growth habit in faba bean (Avila et al. 2006, 2007) (Table 3a). The GAB approaches have been limited till now due to unavailability of such relevant DNA markers; however, the above identified markers linked to agronomically important traits along with additional markers for other important traits in coming days from ongoing mapping projects will help to commence GAB in these pulse crops.

### Harnessing allelic variation through association genetics

Given segregation of only two alleles, the FBL mapping is the most appropriate method for capturing *rare* alleles; however, it lacks *precision* in locating QTLs within the genome (Cavanagh et al. 2008). In contrast to FBL, AM tests non-random association of alleles or LD in a set of diverse and non-related individuals with no extra efforts given to the generation of a large experimental population (Mackay and Powell 2007). In AM, establishing a marker–trait association largely depends on the rate of LD decay. Although not uniform across the whole genome, LD decays at a much higher rate in outbreeding crops compared to self-pollinated species (Yu and Buckler 2006). However, successful instances of LD analyses in various self-pollinated species like barley (Cockram et al. 2010), and subsequently in several species like rice and wheat (see Galeano et al. 2012), offer new prospects for AM-based discovery of important QTL-containing regions in pulses as well.

With increasing availability of large-scale genetic markers in most of the pulse crops, AM would likely be the method of choice for high-resolution QTL discovery. For instance, the AM method was applied to diverse collections from ‘USDA Pea Core’ to examine the associations of various candidate genes with yield/yield-relevant traits and, consequently, the role of some pea homologues of APETALA2 (AP2) and GA 3-oxidase (GA3ox) with regard to yield was revealed (Murray et al. 2009). Kwon et al. (2012) also analysed the marker (SSR, RAPD and SCAR) and phenotyping data in 285 USDA pea core accessions using models such as generalized linear model (GLM) and mixed linear model (MLM) and significant marker–trait linkages were obtained for mineral nutrient concentrations, disease/pest resistance and other important morphological traits.

By estimating genome-wide LD decay in asparagus bean, Xu et al. (2012a) proposed that LD extends up to a long physical distance (~2 cM or 1.86 Mb) in asparagus bean. Besides advocating the existing hypothesis about *unguiculata*–*sesquipedalis* divergence, this investigation provided novel insights such as the role of three specific chromosomes during cowpea domestication. These three LGs (5, 7 and 11) showed markedly different patterns of LD decay between the two cultivar groups, viz. *unguiculata* and *sesquipedalis*. From the trait mapping perspective, this study offered a concrete framework for initiating genome-wide association (GWA)-based dissection of complex traits in cowpea. More recently, Muchero et al. (2013) performed whole-genome scan in a panel of 383 diverse cowpea accessions using 865 SNPs. The MLM approach identified several QTL regions associated with delayed senescence, biomass and yield/yield components. Moreover, the report also provided evidences about the presence of pleiotropic-effect QTLs for stay-green trait in cowpea. Furthermore, QTLs for delayed senescence, drought tolerance and yield were validated in another RIL population (IT93 K-503-1 × CB46). In a similar way, the GWA study involving 171 cowpea accessions confirmed the existence of seed weight-QTLs (*Css 1*-*10*), which were initially detected in eight different RI populations by family-based QTL analysis. Further, most of the underlying QTLs exhibited syntenic relationship with genomic regions controlling seed weight in soybean. Notably, one of the candidate QTLs (*Css*-*3*) colocalized with another QTL known to impart resistance to foliar thrips (*Thr*-*1*) in cowpea, whereas two other QTLs (*Css*-*4* and *Css*-*9*) overlapped with loci governing charcoal rot resistance (*Mac*-*6* and *Mac*-*8*) (Lucas et al. 2013b). The AM approach was also used in lentil for detection of significant QTLs associated with various seed-relevant traits. A set of 140 accessions comprising various breeding lines, cultivars and landraces was genotyped with ~900 GG-based SNPs and subsequently, QTLs were recovered for seed diameter, seed thickness and seed plumpness (Fedoruk 2013).

The confounding effects of population structure or genetic relatedness, however, remain the biggest impediment to AM that often lead to the generation of various spurious associations or *false positives* (Korte and Farlow 2013; Mitchell-Olds 2010; Varshney et al. 2012). This limitation may be overcome through employing GWAS in MAGIC or NAM populations, which are intrinsically devoid of any complex structure (Bandillo et al. 2013; Cook et al. 2012; McMullen et al. 2009; Tian et al. 2011). In this way, multi-parent genetic populations bridge the gaps between FBL and LD-based approaches and hold great potential for high-resolution trait mapping.

### Reverse genetics approaches for gene discovery

Reverse genetics comprises an array of approaches like transgenic-based as well as non-transgenic systems like virus-induced gene silencing (VIGS) and targeting-induced local lesion in genomes (TILLING). To establish a transgenic system the prerequisites are: (1) an efficient and reliable genetic transformation procedure, (2) a reproducible, economically viable and easy-to-use regeneration protocol and (3) an appropriate selectable marker with corresponding selective agent to recover transformants (Popelka et al. 2004; Svabova and Griga 2008). To introduce foreign DNA into plant cells, two techniques, viz. *Agrobacterium*-mediated and direct DNA transfer including electroporation, mircoprojectile bombardment and polyethylene glycol (PEG), have been used in these pulse crops (Eapen 2008; Popelka et al. 2004; Somers et al. 2003). Of all the techniques used for DNA delivery, *Agrobacterium tumefaciens*-mediated transfer has been widely accepted as the standard method in legumes (Atif et al. 2013; Eapen 2008; Somers et al. 2003). Conversely, alternative methods involving direct DNA transfer are known to generate relatively elevated number of chimeras (Chandra and Pental 2003; Popelka et al. 2004). Nevertheless, direct DNA transfer represents the sole method for introducing a foreign gene into organellar genomes (Atif et al. 2013).

In general, the frequency of transformation in pulse crops is considerably low as compared to cereals (Atif et al. 2013; Chandra and Pental 2003; Eapen 2008). For example, some recent genetic transformation experiments have reported frequencies of 3.09–3.6 % in cowpea (Bakshi et al. 2011, 2012), 0.1–1.0 % in pea (Svabova and Griga 2008), 0.9 % in lentil (Chopra et al. 2011) and 0.15–2 % in faba bean (Hanafy et al. 2005). Given the context, Svabova and Griga (2008) considered co-cultivation as a decisive step towards enhancing the transformation efficiency and evaluated the effects of application of various chemicals such as acetosyringone, l-cysteine, dithiothreitol, glutathione, cellulase and pectinase while performing co-cultivation in pea. Previously, Olhoft and Somers (2001) reported a fivefold increase in stable DNA integration by applying l-cysteine to the solid co-cultivation medium in soybean. Besides use of chemical additives, sonication and vacuum infiltration-assisted methods have also been reported to improve the efficiency of genetic transformation in these crops (Bakshi et al. 2011; Chopra et al. 2011).

Furthermore, concerning the mode of regeneration in pulse crops, direct organogenesis (without callus formation) has been preferred over somatic embryogenesis (Atif et al. 2013; Chandra and Pental 2003). However, recalcitrance and genotype-specific response of various pulse crops to these regeneration protocols are other major issues challenging their routine use in transgenic development. To overcome the issue of recalcitrance to regeneration in vitro, Somers et al. (2003) suggested exploring the possibilities of non-tissue culture-based transformation, which avoids labour-intensive culture practices and eventually eliminates other related problems including somaclonal variations (Griga et al. 1995) and differential response of genotypes to regenerate (Tague and Mantis 2006). Recently, Weeks et al. (2008) developed a genotype-independent and marker-free *in planta* transformation system for alfalfa (*Medicago sativa*) with enhanced transformation efficiency (~7 %). Though constant refinements are being made in the transformation systems and regeneration protocols, stable transmission of a foreign gene to subsequent progenies and its predictable expression still remains challenging (Gelvin 2003; Popelka et al. 2004). Nevertheless, the transgenic-based RNA interference (RNAi) technologies have greatly helped in understanding the molecular mechanisms of nitrogen fixation in legumes. For instance, the role of *Rba 2* gene in *Phaseolus*–*Rhizobium* symbiotic relationship was elucidated using RNAi technology with no induction observed for early nodulation genes (Antonio Blanco et al. 2009). In addition to exploring symbiotic nitrogen fixation, RNAi was also used to examine the mechanism of resistance against various biotic constraints in pulses (Bonfim et al. 2007).

The non-transgenic approaches are particularly suitable for legumes, which are not amenable to routine transformation/regeneration protocols (Tadege et al. 2009). One of such powerful and HTP techniques is TILLING, which involves chemical mutagenesis, and a sensitive mutation-detecting instrument, therefore making it amenable to automation. The basic steps followed in TILLING are: (1) generation of a TILLING population, (2) isolation and pooling of DNAs, (3) PCR amplification with gene-specific labelled primers, (4) denaturation and re-annealing followed by hetero-duplex formation, (5) cleavage at mismatch using enzymes like *CEL1* endonuclease and (6) detection of cleaved products using instruments such as LI-COR (Gilchrist and Haughn 2005; McCallum et al. 2000; Tadege et al. 2009). In pea, a global TILLING platform has been developed with two EMS-induced mutant populations from two genotypes: ‘Cameor’ (4,704 M2 lines) and ‘Terese’ (3,072 M2 lines). The ‘Cameor’ population, also referred to as ‘reference TILLING population’, successfully allowed molecular screening of 54 genes (http://www-urgv.versailles.inra.fr/tilling/pea.htm; Dalmais et al. 2008) with the notable mutation detection in the pea methyl transferase 1 gene (*PsMet1*). Further, the efficacy of *Arabidopsis thaliana* mismatch-specific endonucleases (*ENDO1*) to detect mutation in gibberellin 3 beta-hydrolase gene of *P. sativum* was successfully demonstrated in the ‘Terese’ population (Triques et al. 2007). Moreover, an in silico database ‘UTILLdb’ has been set up to enable access to the phenotypic expression and sequence information on mutants (Dalmais et al. 2008). TILLING has also contributed to understanding the function of pea subtilase (*SBT1.1*) and tendril-less (*tl*) genes in controlling seed size and tendril formation, respectively (D’Erfurth et al. 2012; Hofer et al. 2009).

Apart from RNAi and TILLING, VIGS is another reverse genetics technique for discovery and characterization of the causative gene(s). Grønlund et al. (2010) successfully applied VIGS technique in pea to suppress genes that are involved in nitrogen-fixing *Rhizobium* as well as in developmental processes. Similarly, the role of *CHLI* and *CHLD* genes in tetrapyrrole biosynthesis and chloroplast development was examined in pea using the VIGS approach (Luo et al. 2013). Despite some notable achievements of reverse genetics approaches, these methods are not so popular as these are time consuming, very costly and can only be exercised in selected institutions/organizations. Nevertheless, further advancements in technology may provide better implementation of such research experimentations with generation of substantially useful information for further improvement of pulse crops.

## Developing Web tools for community-oriented research

With a deluge of omics information being generated worldwide, easy access to data remains one of the foremost challenges to large-scale integration of omics information into crop improvement (Main et al. 2013). The community-based approach has facilitated the development of several Web interfaces for various pulse crops, allowing storage and ensuing retrieval of data in a very systematic and user-friendly manner (Table 4). These databases offer a comprehensive view of the available genetic resources like mutant stocks/germplasm collections, genomic tools including BACs, BESs, markers, maps, QTLs and transcriptomic resources such as cDNA libraries and ESTs. Moreover, these Web tools integrate several other databases/browsers enabling comprehensive computational analyses for comparative genomics studies. For example, a popular legume Web resource namely Legume Information System (LIS) was developed by the National Center for Genome Resources (NCGR) and the United States Department of Agriculture (USDA), which incorporates several other databases and Web interfaces including SoyBase, CMap and comparative functional genomics browser (CFGB) (Gonzales et al. 2005). In the interest of the pulse research community, it is very essential to keep these websites updated with newer useful information.

**Table 4:** Web tools developed for selected pulse crops

Resource	Link	Content	Reference
Legume Information System (LIS)	http://comparative-legumes.org/	Sequenced genomes, annotations, BACs and BESs, transcriptome assemblies, genetic and comparative maps, primer sequences, etc.	Gonzales et al. (2005)
KnowPulse	http://knowpulse2.usask.ca/portal/	Genetic resources, mapping populations, markers, genotype data with phenotypic assessment of available resources, annotation tools, etc.	Sharpe et al. (2013)
Cool Season Food Legume Genome Database	http://www.gabcsfl.org/main	cDNA libraries, ESTs, genetic markers, maps and genome sequencing information	Main et al. (2013)
BeanGenes	http://beangenes.cws.ndsu.nodak.edu/	Germplasm information, QTLs, pathogen descriptions	McClean (1995)
Cowpea Genespace/Genomics Knowledge Base (CGKB)	http://cowpeagenomics.med.virginia.edu/CGKB/	Genetic markers, gene-space, metabolic pathways, mitochondrial and chloroplast sequences	Chen et al. (2007)
The Cowpea Genomics Initiative (CGI)	http://cowpeagenomics.med.virginia.edu/	Recent advances in cowpea genomics	Chen et al. (2007)
HarvEST:Cowpea	http://harvest.ucr.edu/	EST database with gene function analysis and primer design	Muchero et al. (2009a, b); Close et al. (2011)
PhyMap cowpea	http://phymap.ucdavis.edu:8080/cowpea/	Cowpea physical map assembly and BAC contigs	Close et al. (2011)
URGV TILLING pea database (UTILLdb)	http://urgv.evry.inra.fr/UTILLdb	Mutant collections of pea, tomato and *Brachypodium*	Dalmais et al. (2008)
Pgene	http://data.jic.ac.uk/cgi-bin/pgene/default.asp	Detailed information about *Pisum* genes and mapping populations	Rubiales et al. (2011)
Pea genetic stocks collection	http://www.ars.usda.gov/Main/docs.htm?docid=15144	A comprehensive collection of pea accessions provided by Prof G.A. Marx	Rubiales et al. (2011)

## GAB in pulse crops: advancing from MAS to GS

The establishment of marker–trait associations in these crops has opened new avenues for applying knowledge-based breeding, which focuses on crossing of genotypes and selection of appropriate offspring on the basis of QTL(s)/marker(s) rather than relying entirely on phenotypic expression. Outstanding success stories on the deployment of the marker(s)/QTL(s) in routine breeding programme are available in several crops including rice, maize, wheat, pearl millet and mustard (Gupta et al. 2012a). In case of pulses, a relatively poor genomic infrastructure has prevailed for a long time, which has hampered the initial investments in GAB; however, recent developments in pulses genomics have led to initiation of several MAS projects.

It was the classic work by Karl Sax in common bean, which laid the foundation of modern theory of association between genetic markers and quantitative traits. He examined linkages of size differences with seed coat pattern and pigmentation (Sax 1923). Thenceforth, DNA markers have greatly contributed making MAS an integral component of pulse breeding. The utility of SCAR markers (MahSe2 and C42B) in discriminating *Striga* resistant and susceptible lines was successfully demonstrated in cowpea (Omoigui et al. 2012). In lentil, selection based on markers UBC 227_1290_ (RAPD)/RB18_680_ (SCAR) and OPO6_1250_ (RAPD) associated with *Ascochyta* blight and *Anthracnose* resistance, respectively, allowed identification of genotypes carrying resistance genes to both *Ascochyta* blight and *Anthracnose* (Tar’an et al. 2003a). Similarly, a robust CAPS marker was used for MAS in faba bean and exhibited 100 % accuracy in distinguishing determinate and indeterminate genotypes in the F_2_ population (Verde Bonita’ ×2N52) (Avila et al. 2006). Likewise, indirect selections using SCAR markers (linked with the genes: *zt*-*1* and *zt*-*2*) were successful (accuracy up to 95 %) in discriminating high tannin-containing genotypes from genotypes with zero tannin content (Torres et al. 2010). The CAPS markers associated with low vicine and convicine content are also good candidates for practising MAS against these major anti-nutritional factors (Gutiérrez et al. 2006).

Marker-assisted back crossing (MABC) is the simplest way to introgress QTLs, particularly a finite number of QTL(s)/gene(s) experiencing strong and durable effects on the phenotype (Varshney et al. 2012; Xu et al. 2012b). Alternatively to capture multiple QTLs with smaller effects, the idea of marker-assisted recurrent selection (MARS) was propounded (Ribaut et al. 2010). Given the demerits of phenotypic recurrent selection (RS) like imprecise selection and lengthy breeding cycles, the MARS scheme offers a marker-aided refinement over RS in which selection and inter-mating are based on marker scores (Ribaut and Ragot 2007; Ribaut et al. 2010). Unlike MABC, MARS can be initiated without any prior knowledge of QTLs with the objective of discovering and harnessing the superior QTLs/alleles during the MARS scheme itself (Bernardo and Charcosset 2006). Empirical and simulation results obtained in maize, soybean and sunflower have encouraged the research community to extend MARS scheme to these pulse crops. For example, MARS programmes have been recently initiated in cowpea involving several populations, each derived from two elite parents (Huynh et al. 2014).

Sometimes, introgressed QTLs may not be able to reproduce the expected phenotype due to fresh genetic interactions that are established with the new genetic background (Grandillo and Tanksley 2005). Given the above-mentioned repercussion of QTL–background interactions, the advanced backcross QTL (AB-QTL) scheme was proposed that could facilitate detection as well as transfer of QTLs within the same mapping population. AB-QTL generates new prospects to explore the underutilized genetic variation contained in the CWRs (Tanksley and Nelson 1996). Though widely accepted in cereals like wheat, rice, barley and maize (Grandillo and Tanksley 2005), AB-QTL has not shown significant impacts in pulse crops. Among the various pulse crops, AB-QTL populations have been developed only in few crops like common bean and pigeonpea (Blair et al. 2006; Varshney et al. 2013a). In particular, the CWR-derived populations have great scope in improving crops that have suffered from severe domestication bottlenecks and extremely narrow genetic base in the primary gene pool. Owing to immense variability for domestication forms, pea is considered an excellent system to understand the genetic basis of changes that occurred during the process of domestication. A set of five broad-based genetic populations was established in pea using a wild ancestor (*P. sativum* ssp. *elatius*) and primitive landrace (*P. sativum* ssp. *abyssinicum*), and the investigation revealed important genes/QTLs for domestication-related traits that collectively represent a ‘domestication syndrome’ (Weeden 2007). The pulse crops have fairly less genetic diversity in the cultivated pool and, hence, development of such broad-based genetic populations is a highly desirable strategy to expand the genetic base.

**Fig. 2:**
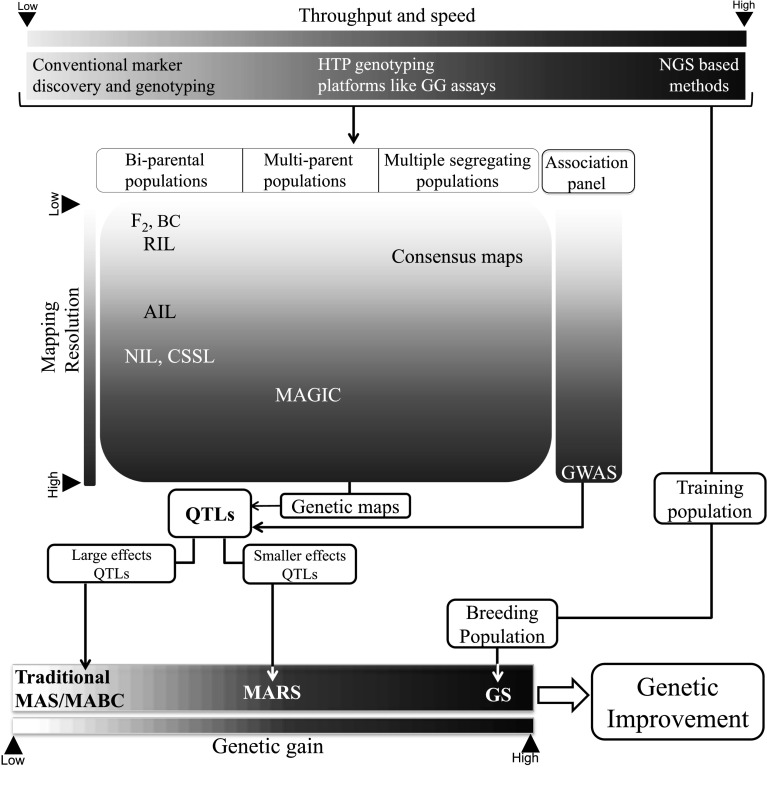
Integrative genomics and breeding approach for accelerated genetic improvement in pulse crops. The figure depicts that methodological shifts in marker discovery/genotyping and QTL mapping strategies have enhanced the throughput and resolution, respectively. Different kinds of mapping populations/association panels are used to establish the gene–trait associations. Concerning introgression of QTLs, MABC aims at transferring limited number of QTLs, while MARS enables accumulation of several QTLs. GS relies entirely on GEBV estimates and these estimates can be employed directly in breeding population for selection of superior genotypes. While practising GAB, the magnitude of genetic gain increases in the following order: MABC < MARS < GS

In recent years, noteworthy changes were experienced in the throughput and accuracy of several genotyping platforms and NGS systems (Xu et al. 2012b). In parallel, a continued search for more efficient and high-throughput molecular breeding methods has resulted in the introduction of a novel approach for genetic improvement, in which selections are made on the basis of *genomic estimated breeding values* (GEBVs) (Meuwissen 2007). The GEBVs are calculated using genome-wide DNA marker information and choosing worthy individuals based on GEBV is referred to as genomic selection (GS) (Heffner et al. 2009; Meuwissen et al. 2001). In GS, high-density genotyping and phenome-level phenotyping are performed for *training population*. On the other hand, the *candidate population* (another component of GS) is used for genotyping only and eventually for selecting the superior individuals (Nakaya and Isobe 2012). As evident from the above description, no additional phenotyping is required for the candidate population. Hence, GS efficiently exploits the high-density marker data available at a reasonable cost, and at the same time it dramatically reduces the experimental cost by circumventing the need for repeated phenotyping (Heffner et al. 2009; Xu et al. 2012b). Keeping the recent genomics advances in view, a holistic approach for improvement of pulse crops has been illustrated in Fig. 2.

## Summary and perspectives

To realize the enormous potential of genomic tools and technologies, it is essential that these tools should become an integral part of regular pulse breeding programmes so that all the accumulated resources and genomic knowledge could be translated into improved cultivars. The wide applicability of MAS has already been demonstrated in cowpea and pea, while in the case of lentil and faba bean it is in infancy stage. However, one encouraging fact is that exceptional progress has already been made in generating ample genomic resources in all the major pulse crops. To this end, the availability of reference genome sequences opens an exciting future for genomic-assisted pulse improvement. Though the prices of HTP genotyping and sequencing have come down to an affordable level, phenotyping of complex traits remains cumbersome, cost prohibitive and environmentally sensitive. Therefore, there is a compelling need to deploy modern molecular breeding methods such as MARS and GS that are able to reap maximum benefits from declining genotyping prices, while demanding the least (one-time) phenotyping. In addition, the recently developed NGS-based methods like WGRS/GBS/RADseq would efficiently extract valuable information from complex mapping resources such as MAGIC or NAM. Besides high-resolution QTL mapping, nearly homozygous MAGIC lines have direct implications in variety development (see Bandillo et al. 2013). These advanced molecular breeding approaches thus represent the next generation of MAS that would greatly assist breeders to strengthen as well as reorient the pulse breeding programmes.
